# Classification of upper limb center-out reaching tasks by means of EEG-based continuous decoding techniques

**DOI:** 10.1186/s12984-017-0219-0

**Published:** 2017-02-01

**Authors:** Andrés Úbeda, José M. Azorín, Ricardo Chavarriaga, José del R. Millán

**Affiliations:** 1 Brain-Machine Interface Systems Lab, Miguel Hernández University, Av. de la Universidad, S/N, Elche, 03202 Spain; 2 Defitech Chair in Brain-Machine Interface (CNBI), École Polytechnique Fédérale de Lausanne (EPFL), Chemin des Mines 9, Geneva, CH-1202 Switzerland

**Keywords:** Brain-computer interface, Electroencephalography, Linear decoding, Upper limb movements, Center-out reaching tasks

## Abstract

**Background:**

One of the current challenges in brain-machine interfacing is to characterize and decode upper limb kinematics from brain signals, e.g. to control a prosthetic device. Recent research work states that it is possible to do so based on low frequency EEG components. However, the validity of these results is still a matter of discussion. In this paper, we assess the feasibility of decoding upper limb kinematics from EEG signals in center-out reaching tasks during passive and active movements.

**Methods:**

The decoding of arm movement was performed using a multidimensional linear regression. Passive movements were analyzed using the same methodology to study the influence of proprioceptive sensory feedback in the decoding. Finally, we evaluated the possible advantages of classifying reaching targets, instead of continuous trajectories.

**Results:**

The results showed that arm movement decoding was significantly above chance levels. The results also indicated that EEG slow cortical potentials carry significant information to decode active center-out movements. The classification of reached targets allowed obtaining the same conclusions with a very high accuracy. Additionally, the low decoding performance obtained from passive movements suggests that discriminant modulations of low-frequency neural activity are mainly related to the execution of movement while proprioceptive feedback is not sufficient to decode upper limb kinematics.

**Conclusions:**

This paper contributes to the assessment of feasibility of using linear regression methods to decode upper limb kinematics from EEG signals. From our findings, it can be concluded that low frequency bands concentrate most of the information extracted from upper limb kinematics decoding and that decoding performance of active movements is above chance levels and mainly related to the activation of cortical motor areas. We also show that the classification of reached targets from decoding approaches may be a more suitable real-time methodology than a direct decoding of hand position.

## Background

The possibility of bypassing neuromuscular control or, in other words, activating an alternative pathway for the brain to act upon the environment, has triggered a fascinating field of research. Brain-Machine Interfaces (BMIs) are devices aimed at translating subjects’ brain activity into commands [1, 2]. They enable people with motor disabilities to interact with their environment in a completely new way [3]. They have been used alone or in combination with other systems, such as Functional Electrical Stimulation (FES), prosthetic arms or hand orthoses, to restore grasping functionalities in subjects with Spinal Cord Injury (SCI), where the loss of motor function is permanent [4]. Moreover, BMIs have become a promising tool in rehabilitation procedures where patients have movement limitations or difficulties to control their limb function [5–7]. Particularly, motor impairment after stroke is one of the main causes of permanent disability. This section of the population usually suffers from upper limb movement limitations and the recovery of the arm movement is often variable and incomplete [8]. This recovery is crucial in order to perform activities of the daily life, so the use of BMIs during the rehabilitation may be a key factor of improvement [3].

Currently, one of the main challenges of BMIs is to characterize and decode upper limb kinematics from brain signals. Up to now, decoding approaches were mainly centered on intracortical recordings, usually performed in non-human primates, where arrays of microelectrodes are implanted directly in the motor cortex. In some studies, the motor cortical activity of monkeys was used to perform reaching and grasping activities with a robot arm [9], or to perform three dimensional movements that included force grasping for self-feeding using a mechanical device [10]. Invasive approaches have been successfully used in people with motor disabilities to perform reaching and grasping tasks [11, 12]. Less invasive procedures such as electrocorticography (ECoG) have also been used to decode two-dimensional arm trajectories [13] and different types of grasping [14]. Despite their potential, invasive approaches require surgery, which limits their use. In this respect, non-invasive methods can compensate the drawbacks of intracortical recordings. Some studies have used magnetoencephalographic (MEG) signals to predict hand movements to perform 2D trajectories [15]. MEG signals have also been used in combination with electroencephalographic (EEG) signals to discriminate between different center-out movements [16]. However, the low signal-to-noise ratio of EEG signals makes it difficult to decode hand movement trajectory.

Recent works suggest that it is possible to decode hand or arm kinematics (position and velocity) from slow cortical potentials (SCPs), i.e., EEG signals oscillations below 2 Hz [17–20]. To that end, multidimensional linear regression models are applied to the data. However, it has been pointed out that this methodology has the risk of overestimating the decoding performance due to the mathematical properties of linear regression between signals in the same frequency range (in this case, slow arm movements and slow cortical potentials) [21]. Furthermore, this later study states that decoding accuracies achieved with SCPs are not above chance level. A previous work also proposed the use of multidimensional linear regression as the decoding method to control a cursor [22]. It reports that it is possible to accomplish a two-dimensional control of this cursor with performance levels comparable to those of invasive BMI systems. In their study, the decoding models had to be recalibrated to include a scaling factor due to the fact that the correlation metric is invariant to scale. Again, the way of how these results are assessed is still a matter of discussion [22–24], so it is necessary to gather further evidence of the real possibilities of decoding arm trajectories from EEG SCPs. In this regard, some studies have suggested the introduction of electromyographic information (EMG) into this decoding procedure [25] or even the use of muscle synergies activation coefficients extracted from this EMG information [26].

In this paper, we compare several results obtained by applying linear regression techniques to decode upper limb kinematics from EEG signals using a center-out reaching approach. We analyze arm movements using the same decoding approach proposed in previous studies [17]. The results show that arm movement decoding was significantly above chance levels. Moreover, we have analyzed passive arm movements using the same protocol to study if the neural information for decoding was related to the execution of movement, instead of being linked to proprioceptive feedback. The final decoding performance obtained from our study suggests that, although neural correlates can be decoded when performing upper limb movements, the decoding accuracy may not be high enough to perform a real-time control of a cursor in a 2D environment and the method is also subject to the scaling limitations. As a consequence, we also evaluated the classification of the reached targets which yielded a very high classification accuracy. From these findings, it appears that the a classification of reached targets from decoding approaches may be a more suitable real-time methodology for rehabilitation purposes (where movements are often repetitive) than a direct decoding of hand position.

## Methods

### Experimental tests

**Fig. 1:**
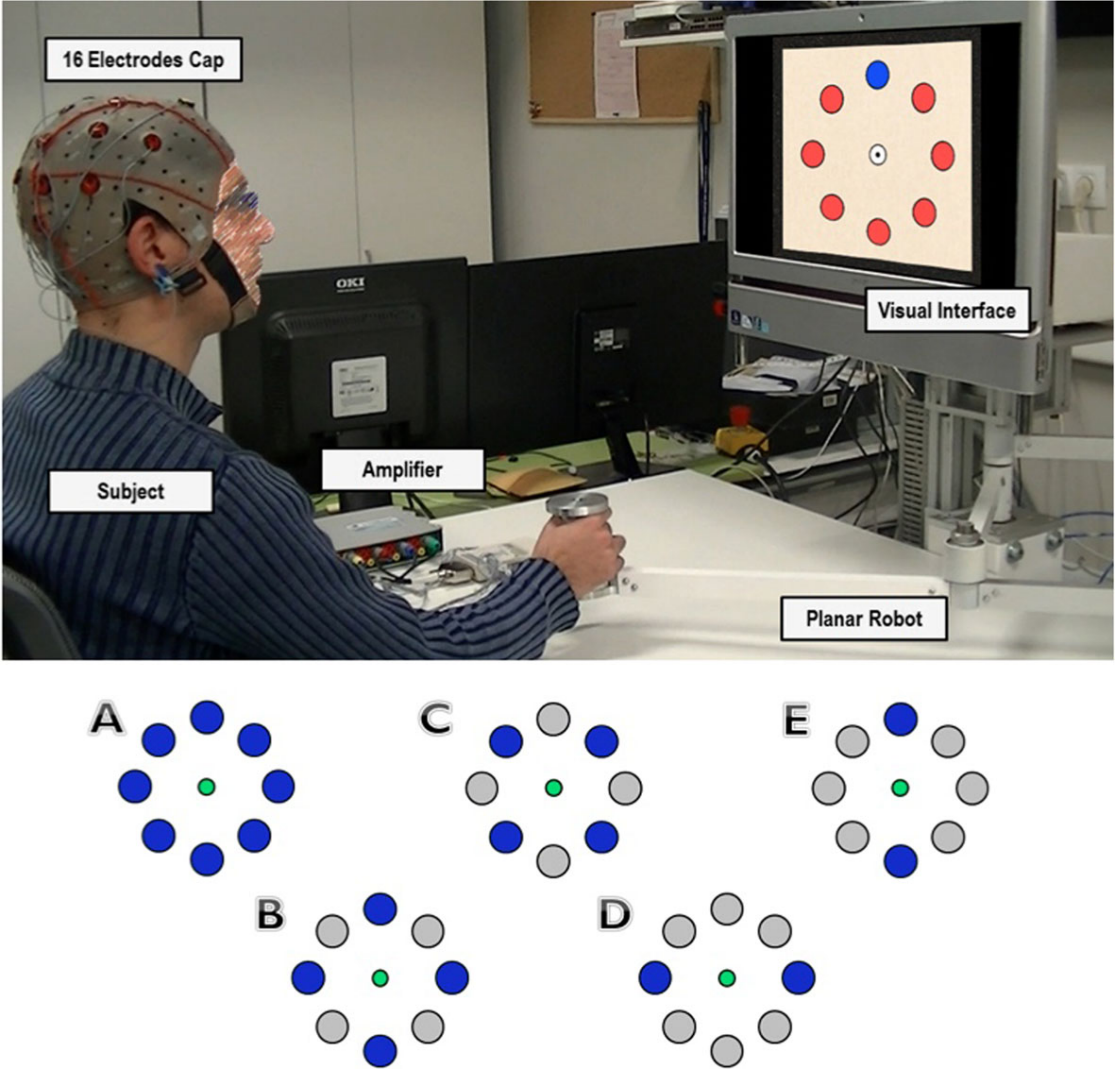
Experimental Environment. *Top*, experimental setup. The subject performs the center-out movements in front of a screen showing the cursor and target locations. The subject is asked to reach the highlighted targets with the planar manipulandum. *Bottom*, different configuration of possible targets have been analyzed to compare the performance of target decoding. Configuration *A* covers all the targets shown. For configurations *B* and *C*, 4 targets are taken into account in the analysis. Finally, configurations *D* and *E* correspond to a discrimination between two opposite target directions
- *Passive center-out movement*: subjects are asked to passively grasp the planar manipulandum while the researcher operates it. The experimental tests are the same as with the active center-out movement. Subjects carried out 5 runs in which 40 targets were randomly highlighted (around 3 minutes per run). All reaching positions were equally highlighted (each of them 5 times per run). 5 able-bodied subjects (C1-C5)(25.2±2.6 year-old) performed the tests. Only one subject performed the experiments for both active (B1) and passive (C1) movements.

The experimental tests are based on a center-out protocol in which subjects sat in front of a computer screen where a cursor moves from a central position to several targets equally distributed around it (see Fig. 1, top). EEG signals were recorded along with the position and velocity of the cursor. Two different experiments were performed:

- *Active center-out movement*: subjects control the cursor movement using a planar manipulandum (see Fig. 1, top). The goal is to reach the target that is randomly highlighted on the screen. The subject must reach it and then return to the central position. Targets are distributed around this central position in a circumference with a radius of 10 cm. Each time a target is reached or the cursor enters the central position, a waiting period of 400 ms is introduced. Each subject executed 10 runs in which 40 targets were randomly highlighted (around 3 minutes per run). All reaching positions were equally highlighted (each of them 5 times per run). 5 able-bodied subjects (B1-B5)(26.4±3.1 year-old) performed the tests. 16 electrodes were recorded distributed over the central and parietal cortex, where a higher activity related to arm movements is expected. The equipment used was the gUSBamp (g.Tec, GmbH, Austria) with a sampling frequency of 1200 Hz. The reference was placed on the right earlobe and ground was placed on the AFz position.

EEG human recordings used in this study have been approved by the ethics committee of the Miguel Hernández University of Elche, Spain. Written consent according to the Helsinki declaration was obtained from each subject.

### Preprocessing

First, cursor kinematics were resampled to match EEG signals. EEG signals were visually inspected to reject blinks, and frontal channels were discarded to diminish ocular artifacts. For this reason, the same 16 electrodes were considered for the analysis of all conditions: FC5, FC1, FC2, FC6, C3, Cz, C4, CP5, CP1, CP2, CP6, P3, Pz, P4, PO3 and PO4. According to previous literature, neural correlates of movement kinematics are mainly found in SCPs above 0.1 Hz [27]. As a consequence, EEG signals were band-pass filtered with a zero-phase 4th-order Butterworth filter between 0.1–2 Hz. For comparison purposes they were also filtered between 8–12 Hz, 14–30 Hz and 0.1–40 Hz, to estimate the amount of information present in each frequency band, similar to the study performed by Antelis et al. [21]. Cursor kinematics (position and speed) were also low-pass filtered with a zero-phase 4th-order Butterworth filter below 2 Hz. Finally, for each run, EEG data from each electrode *i* were standardized by subtracting, for each time sample (*t*), the mean ($\bar {V}_{i}$) of the signal and dividing the result by the standard deviation (*S* *D*_*Vi*_) as shown in (1).

1 $$ EV_{i}[\!t]=\frac{V_{i}[\!t]- \bar{V}_{i}}{SD_{Vi}}   $$

### Decoding

A multidimensional linear regression was applied to decode kinematics from EEG signals,

2 $$ x\left (t \right)=a+\sum_{n=1}^{N}\sum_{k=0}^{L}b_{nk}S_{n}\left (t-k \right)   $$

where *x*[ *t*] is the kinematics state (position and velocity) at time *t* and *S*_*n*_ is the signal from channel *n*. *L* corresponds to the number of lags and *N* to the number of channels. The decoding parameters, *a* and *b*, were estimated using a cross-fold validation for both the active movement condition (ten folds) and the passive movement condition (five folds). The values for the parameters *L* and *N* are: *L*=10 (around 80 ms of signal) and *N*=16 (central and occipital electrodes uniformly distributed).

To simplify the process, the matrix form of (2) has been used as follows:

3 $$ X[\!4\times1]=B[\!4\times NF]*S[\!NF\times1]+A[\!4\times1]   $$

where *X* is the kinematic state [ *P* *x* *P* *y* *V* *x* *V* *y*]^′^, *B* is the transformation matrix, *S* is the features array, *A* is the scale matrix and *NF* is the number of features used which depends on the time lag *L* and the number of channels *N* (*N* *F*=*L*∗*N*+1).

### Analysis

#### Movement profiles

We report the speed profiles (mm/second) for each subject and movement condition (active and passive movements). To that end, the average speed for each point in the trajectory (from the central position to the corresponding target) has been computed for each reaching movement, normalized in length and averaged between all trials for each subject and condition. Speed was considered negative when the direction of movement was negative regarding the considered axis. For instance, this means that when the subject was approaching horizontally to a target on his/her left, his/her speed was computed as a negative value, and when he/she was moving to the opposite direction, speed was computed as a positive value.

#### Continuous decoding

For the continuous decoding, the matrices B and A in (3) were obtained using a cross-fold validation (10 folds). For each fold, the training data was used to compute the decoding matrices that are then applied to the test data to obtain the decoded kinematics. We computed the Pearson correlation coefficient between the real and decoded kinematics for each testing fold and reported the performance in terms of average correlation. The results have been compared for different ranges of frequencies (0.1–2 Hz, 8–12 Hz, 14–30 Hz and 0.1–40 Hz). Additionally, shuffled and random data have been used as input to assess if the decoding accuracy was above chance levels. Shuffled data was obtained by randomly mixing target labels of real data and the associated kinematics to keep the temporal structure of the EEG signals in a way equivalent to [21, 28, 29]. Random data was generated as a standard uniform noise with the same size of real input data. Both shuffled and random data were filtered and standardized in the same way as the actual experimental data. Random and shuffled data decoding coefficients were computed 1000 times to avoid chance effects due to the stochastic nature of the process.

#### Classification of reached targets

We evaluate the possibility of classifying reaching movements towards a particular target by analyzing EEG signals in the frequency range (0.1–2 Hz). Only SCPs have been taken into account as the continuous decoding shows non-significant results in other bands (see section Results - Continuous Decoding). To that end, EEG signals and kinematics were manually segmented into blocks for each center-out movement and labeled with the corresponding target. First, the trajectory of the cursor was decoded for each movement block (from the vectors of decoded X and Y positions) and, then, a straight line was fitted using the obtained trajectory and compared to the angular position to each target to infer the movement direction. This classification was performed using a cross-fold validation for 5 different target configurations (see Fig. 1, bottom). The movement workspace was divided into sectors depending on the configuration of targets. For example, for two targets, the workspace was divided into two sectors and the estimated trajectory orientation was assigned to the nearest target. As before, shuffled and random data were used to estimate chance levels.

We also assessed the performance through the estimation of the classification confusion matrices and the information transfer rates. Firstly, confusion matrices have been computed for each configuration and subject to show the extent of misclassification. Secondly, Information Transfer Rates (ITRs) have been computed for the average classification rates obtained by each subject for the different target configurations according to the following equation (for further information see [30]):

4 $$ ITR=log_{2}N+Plog_{2}P+(1-P)log_{2}\frac{1-P}{N-1}  $$

where N is the number of classified targets and P is the accuracy of the classification

ITR values have been plotted over the ITR curves obtained for 2, 4 and 8 classified targets to better show the performance of each subject.

## Results

### Movement profiles

**Fig. 2:**
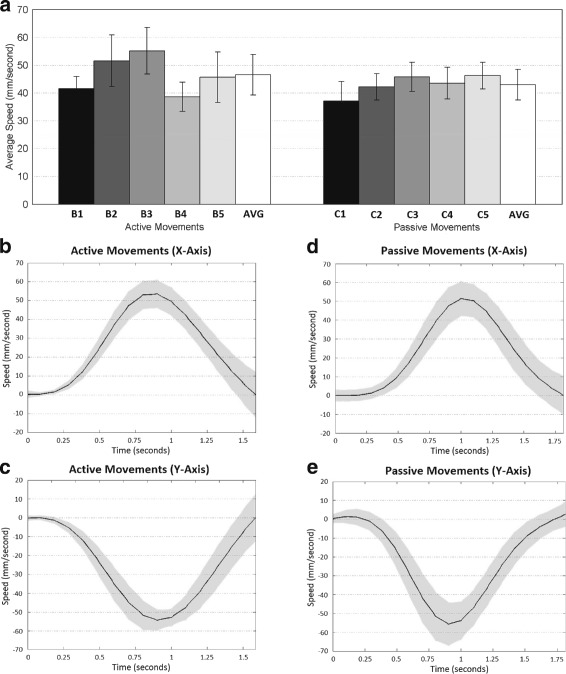
Movement Profiles. Average speed (mm/s) profiles for active and passive movements (**a**). Average time courses (mean ±STD) of the hand X and Y speed (mm/s) for active (**b**-**c**, Subject B1) and passive (**d**-**e**, Subject C1) movements. Speed sign is related to the direction of movement regarding the axis

Figure 2 a reports the average speed of the reaching movements for the active and passive conditions. It shows comparable velocities for both conditions (averaged: 46.49 ± 9.73 mm/s for the active movements and 42.93 ± 6.44 mm/s for the passive movements). For the passive experiments, the same researcher performed the movements for all subjects, which may explain the reduced variability with respect to the active condition where subjects performed the movements by themselves.

Figure 2 b–e shows the average time courses of the X and Y hand speed for an exemplary subject (B1, active movements, and C1, passive movements) and direction (bottom-right target) showing the expected initial acceleration and final deceleration for both conditions.

### Continuous decoding

**Fig. 3:**
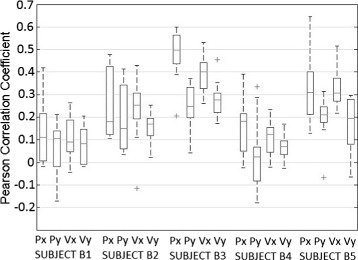
Continuous decoding performance. Decoding performance of center-out trajectories for the frequency band 0.1–2 Hz. The boxplot represents the Pearson correlation coefficient obtained after computing a cross-fold validation between all runs (*n*=10). On each box, the central mark is the median, the edges of the box are the 25th and 75th percentiles, the whiskers extend to the most extreme datapoints which are considered not outliers, and the outliers are plotted individually. For each subject (1–5) the graph shows results for position (Px and Py) and velocity (Vx and Vy)

The Pearson correlation coefficient has been obtained after computing a cross-fold validation between all runs for each subject. Figure 3 shows the Pearson correlation coefficients obtained while performing center-out movements when decoding signals in the frequency band 0.1–2 Hz. The results show high decoding correlations (Fig. 3). Particularly, subjects B3 and B5 obtain the best decoding accuracy with some components reaching a value of 0.5.

**Fig. 4:**
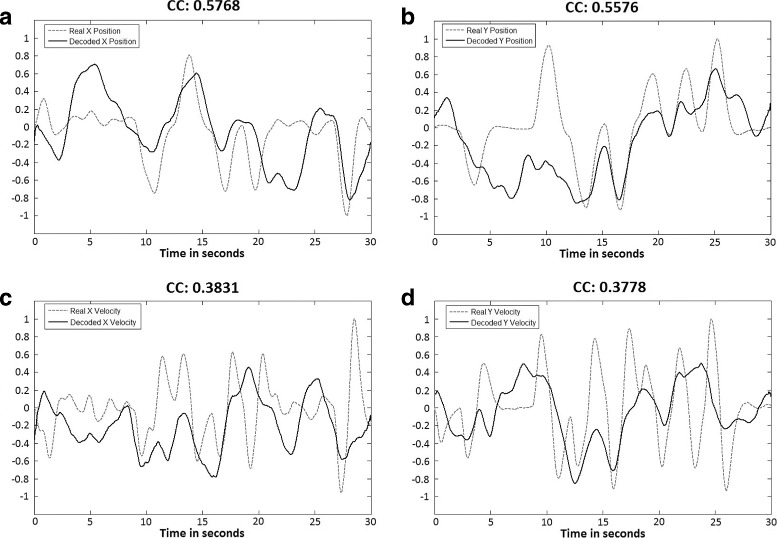
Example of decoded kinematics. Continuous decoding of kinematics using the linear regression decoding method (Subject 3 - Active Center-out Movement). The grey dotted line represents the real performed movement. The continuous black line represents the decoded kinematics (**a** X Position, **b** Y Position, **c** X Velocity, **d** Y Velocity). The correlation coefficient (CC) obtained from the correlation of both signals is also shown

Figure 4 shows an example of 30 s of kinematic reconstruction (2D position and velocity) for one of the subjects performing active movements. In this particular example, decoding coefficients above 0.5 show an accurate reconstruction of the performed trajectories (X Position and Y Position). When the decoding correlation decreases (X Velocity, Y Velocity), the reconstructed signal preserves its tendency but reduces its accuracy.

**Fig. 5:**
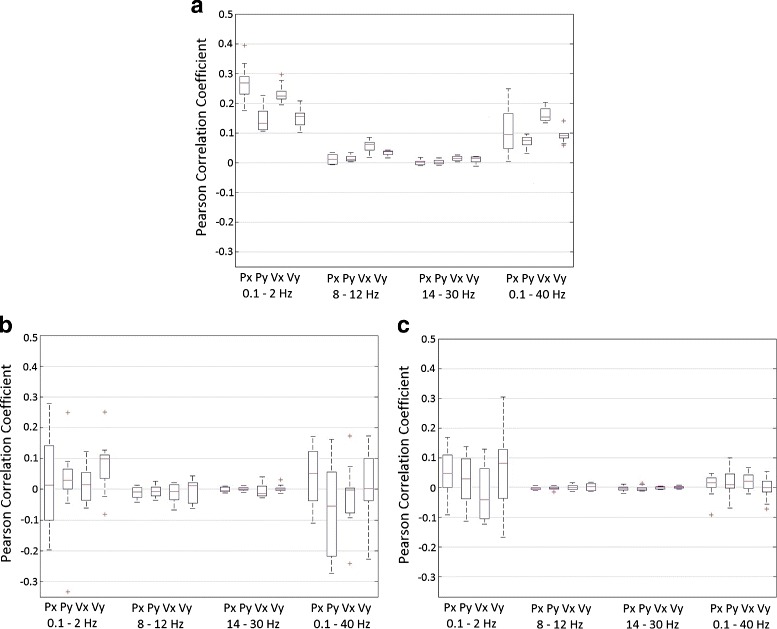
Frequency analysis. Comparison between different frequency bands: 0.1–2 Hz (low frequencies), 8–12 Hz (alpha band), 14–30 Hz (beta band) and 0.1–40 Hz. The boxplot represents the Pearson correlation coefficient obtained after computing a cross-fold validation between all runs (*n*=10) for each subject and then averaged between subjects (*n*=5). On each box, the central mark is the median, the edges of the box are the 25th and 75th percentiles, the whiskers extend to the most extreme datapoints which are considered not outliers, and the outliers are plotted individually. Position (Px and Py) and velocity (Vx and Vy) are shown for different experimental data: center-out movements (**a**), shuffled data (**b**) and random data (**c**)

Previous studies have claimed that upper limb kinematics are better reconstructed from low frequency EEG signals [17, 19, 21]. We tested this hypothesis by analyzing the decoding performance using the signal in four different frequency bands: 0.1–2 Hz (SCPs), 8–12 Hz (alpha band), 14–30 Hz (beta band) and 0.1–40 Hz (Fig. 5). In agreement with these studies, our analysis showed that decoding correlations of higher frequency bands were close to zero and that the low frequency band (0.1–2 Hz) yielded the best decoding accuracies (Fig. 5). Decoding performance using SCPs was slightly but not significantly above results obtained with a broader frequency band (0.1–40 Hz) that includes the irrelevant higher frequencies.

**Fig. 6:**
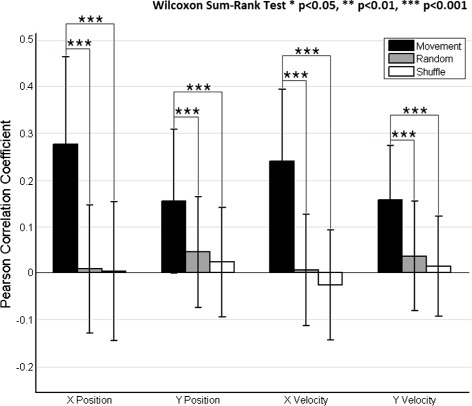
Continuous decoding significance. Decoding performance of center-out trajectories comparing different experimental data: active center-out movement, shuffled data and random data. The Pearson correlation coefficient (mean ± STD) is obtained after computing a cross-fold validation between all runs (*n*=10) and then averaged between subjects (*n*=5). The graph shows results for position (Px and Py) and velocity (Vx and Vy) and reflects differences of active center-out movement versus random and shuffled data. The stars represent significant differences with respect to random and shuffle conditions

To estimate the significance of our findings, the decoding approach was tested with random and shuffled data and compared with the results for active movement (Fig. 6). Active movement was decoded significantly above chance level for all kinematic components (*p*<0.001, Wilcoxon Sum-Rank Test)(Fig. 6). Also, the decoding performance of error and shuffle conditions was not significant (*p*>0.05, Wilcoxon Signed-Rank Test). These findings differ from a previous study [21], where the correlations and normalized errors of the results of real models were not statistically different from shuffled and random models, but are similar to what is obtained in several works related to the topic [20, 28, 29]. This discrepancy could be due to the nature of the experimental data or the way EEG data were processed. However, the results obtained in most of the previous works suggest that decoding performance is significant when linear decoders are applied to slow cortical potentials.

### Classification of reached targets

**Fig. 7:**
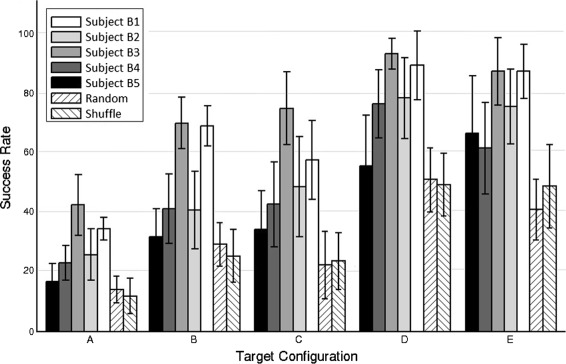
Classification performance. Classification performance of center-out trajectories for active center-out movements. The barplot represents the success rate (mean ±STD) of targets correctly classified obtained after computing a cross-fold validation between all runs (*n*=10). For each subject (1-5) the graph shows results for all the different target configurations (as shown in Fig. 1)

Figure 7 shows the success rate of targets correctly classified after computing a cross-fold validation between all runs recorded for center-out movements. For each subject the graph shows the five different target configurations proposed (Fig. 1, bottom). The results yield a high performance for all the configurations (averaged: 29.0% ±11.8% for configuration A, 51.3% ±19.2% for configuration B, 52.3% ±20.5% for configuration C, 79.6% ±15.9% for configuration D and 75.6% ±17.0% for configuration E). As expected, the performance of each subject in the decoding is consistent with the results in the continuous case. Unsurprisingly, subjects B3 and B5, who obtained the best decoding accuracies in the continuous approach, also had the highest success rates. The success rate obtained in the classification of two targets (configurations D and E) is particularly remarkable (subject B3, 93.0% ±6.7% and subject B5, 89.0% ±11.0% for configuration D; and subject B3, 88.0% ±11.3% and subject B5, 87.0% ±9.4% for configuration E).

**Fig. 8:**
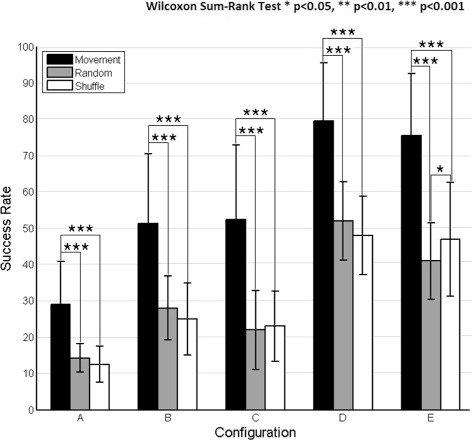
Classification significance. Classification of center-out trajectories comparing different experimental data: center-out movement, shuffled data and random data. The success rate of targets correctly classified (mean ±STD) is obtained after computing a cross-fold validation between all runs (*n*=10) and then averaged between subjects (*n*=5). Each graph shows results for all the different target configurations: A-E (see Fig. 1) and reflects differences of center-out movement versus random and shuffled data

Theoretically, chance level for configuration A (8 targets) should be 12.5%, for configurations B and C (4 targets) 25%, and for configurations D and E (2 targets) 50%. However, as the number of trials is small, these levels may not be representative. As a consequence, the classification of targets was computed for shuffled data and random data in the same way as for the continuous decoding and compared with active movement results (Fig. 8). The results show that the decoding of active movements was significantly above chance level for all configurations (*p* <0.001, Wilcoxon Sum-Rank Test).

**Fig. 9:**
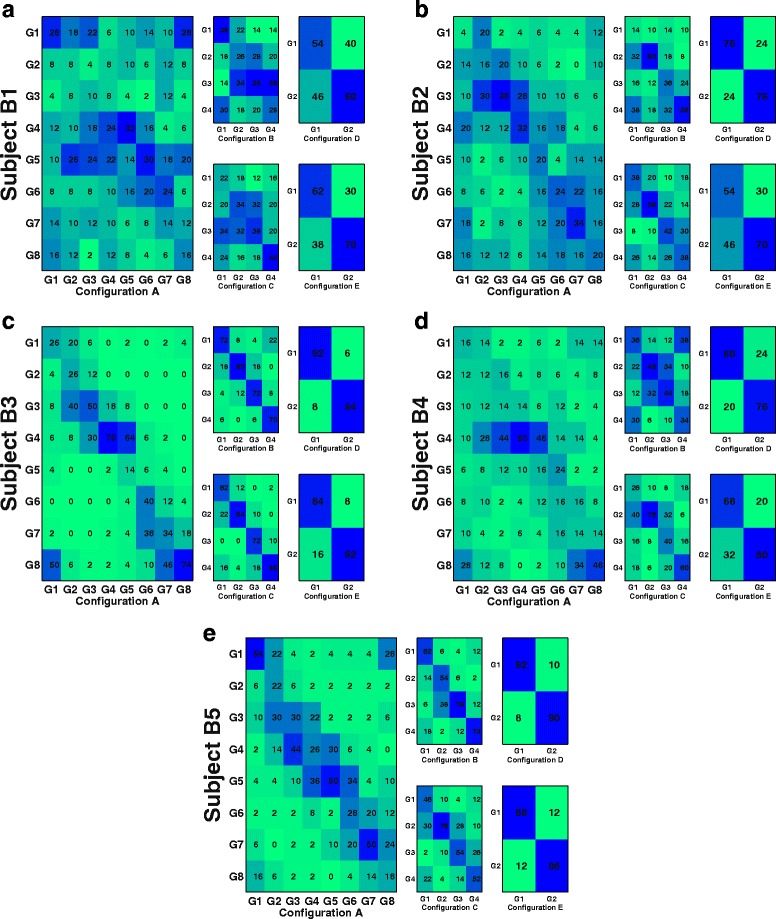
Misclassification rates. Confussion matrices showing classification performance of center-out trajectories for active center-out movements taking into account neighboring classification rates. For each subject (*B1*–*B5*), confusion matrices are shown for all five different target configurations (*a*–*e*). Values are color coded from green to blue (0 to 100%, respectively)

**Fig. 10:**
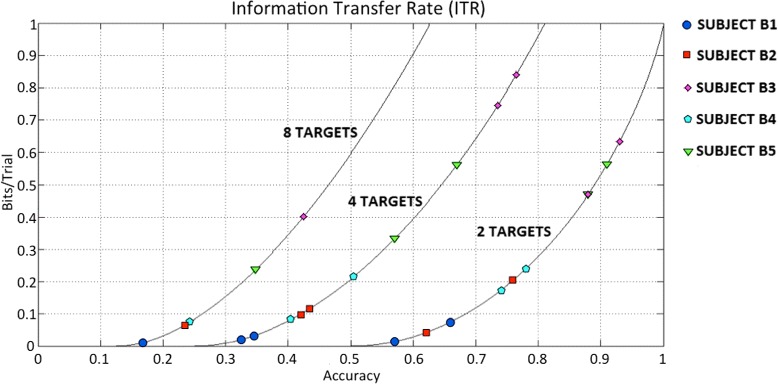
Information Transfer Rates (ITRs). ITRs were computed from average classification rates for all subjects (B1-B5) and number of targets. Base curves represent the whole range of ITR for 2, 4 and 8 classes (targets)

Confusion matrices show that misclassification is mainly focused on the targets closest to the classified target (Fig. 9), suggesting that the classification method is quite robust. This is particularly visible in subjects B3 and B5, who obtained the best decoding accuracies. Consistently, when analyzing information transfer rates (ITRs), subjects B3 and B5 obtain the highest ITRs (Fig. 10). Rates are remarkably high (over 0.5 bits/trial) for configurations B to E. For the remaining subjects and, in general, for configuration A (8 targets), ITR is usually lower.

### Decoding passive movement

The results obtained from the decoding of active center-out movement were significantly above chance level. One possible explanation for these results is that decoding is driven by the influence of proprioceptive sensory feedback while reaching each of the targets instead of reflecting neural correlates of motor intention. To study the influence of afferent feedback in the decoding, we performed a second experiment using passive movements. This new data set was then analyzed the same way as the previous data (decoding of low frequency components 0.1–2 Hz).

**Fig. 11:**
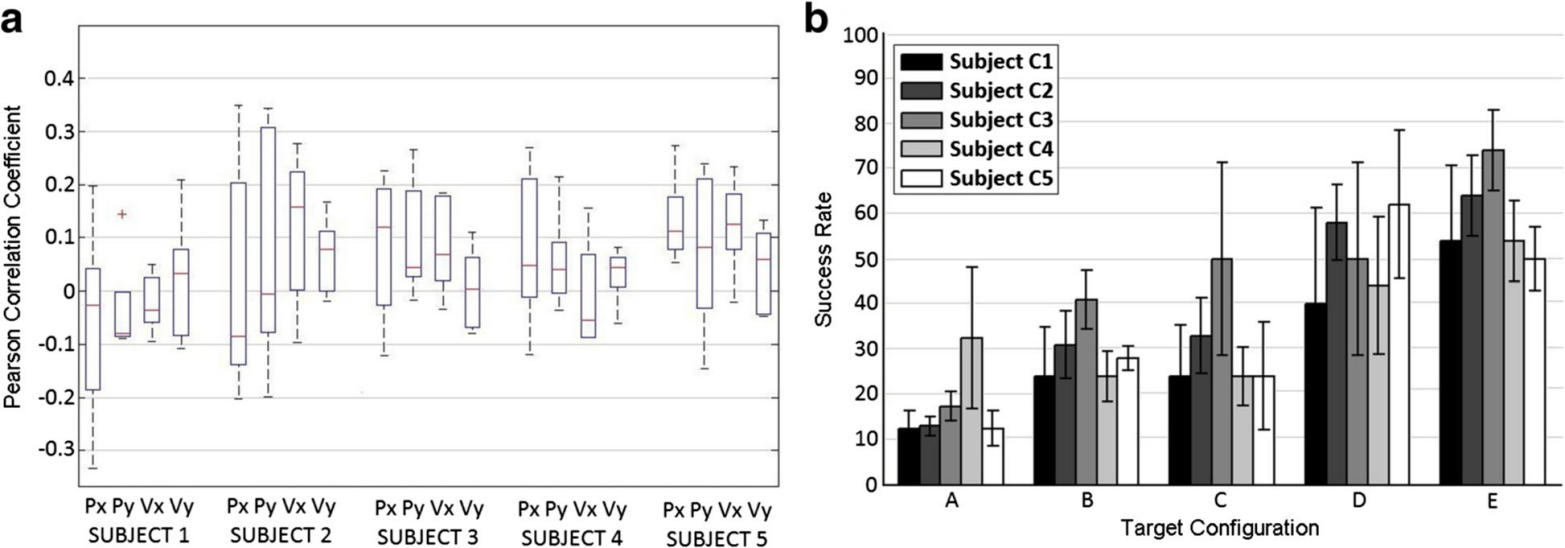
Passive movements decoding. Continuous decoding of center-out trajectories (**a**) and classification of reached targets (**b**) for passive center-out movement. **a** represents the Pearson correlation coefficient (mean ±STD) obtained after computing a cross-fold validation between all runs (*n*=5). On each box, the central mark is the median, the edges of the box are the 25th and 75th percentiles, the whiskers extend to the most extreme datapoints which are considered not outliers, and the outliers are plotted individually. For each subject (1-5) the graph shows results for position (Px and Py) and velocity (Vx and Vy). **b** represents the success rate of targets correctly classified (mean ±STD) obtained after computing a cross-fold validation between all runs (*n*=5). For each subject (1-5) the graph shows results of five different target configurations (see Fig. 1)

**Fig. 12:**
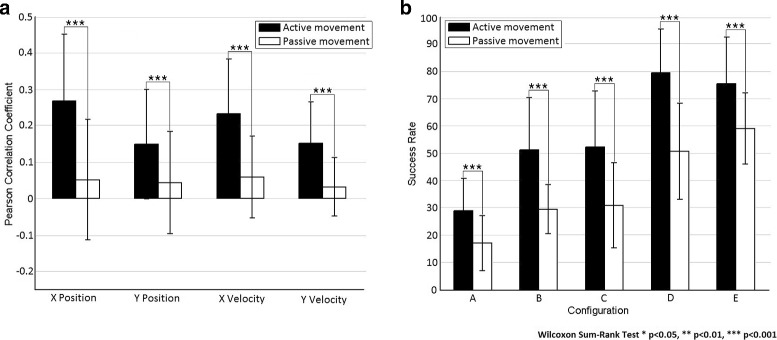
Passive vs active decoding. Continuous decoding of center-out trajectories (**a**) and classification of reached targets (**b**) comparing active center-out movement and passive center-out movement. For the continuous decoding, the Pearson correlation coefficient (mean ±STD) is obtained after computing a cross-fold validation between all runs (*n*=10 for active and *n*=5 por passive) and then averaged between subjects (*n*=5). The results for position (Px and Py) and velocity (Vx and Vy) are displayed. For the classification of reached targets, the success rate of targets correctly classified (mean ±STD) is obtained after computing a cross-fold validation between all runs (*n*=10 for active and *n*=5 por passive) and then averaged between subjects (*n*=5). The results of five different target configurations (see Fig. 1) are displayed

Figure 11 a shows the Pearson correlation coefficient obtained while performing passive center-out movements (continuous approach) and Fig. 11 b shows the success rate of targets correctly classified (classification approach). In both cases, performance was not above chance level (*p*>0.05, Wilcoxon Sum-Rank Test), supporting the hypothesis that EEG slow cortical potentials do carry significant information related to the execution of active center-out movements and proprioceptive feedback is not enough to decode upper limb kinematics. The significance of neural activity during active center-out movements is illustrated in Fig. 12 showing that the decoding accuracy was always significantly higher than for passive movements for all the kinematic components (X Position, Y Position, X Velocity and Y Velocity)(*p*<0.001, Wilcoxon Sum-Rank Test, Fig. 12 a) and the success rate was significantly above the levels of passive movements for all configurations (*p*<0.001, Wilcoxon Sum-Rank Test, Fig. 12 b).

## Discussion

This paper contributes to the assessment of the use of linear regression methods to decode upper limb kinematics from EEG signals. Previous work states that it is possible to decode hand or arm kinematics (position and velocity) from slow cortical potentials, i.e., EEG signals below 2 Hz [17–20]. However, these results may have been misinterpreted due to the inherent properties of linear regression methods, particulary, when comparing EEG signals with the same frequency range as the decoded kinematics [21]. To confirm or reject this conclusion, we have applied a similar methodology to experimental data during the performance of active and passive center-out movements in a two dimensional space.

As previously reported [17, 21, 22], low frequency bands (0.1-2 Hz) concentrate most of the information extracted from upper limb kinematics decoding. According to [21], as slow cortical potentials and the decoded kinematics are sinusoid-like, the correlation of this kind of signals with equal amplitudes and small time-shifts is higher at these low frequencies [21]. This can lead to an overstimation of the decoding performance not related to discriminant modulations of neural activity. Our results and the experimental protocols we have explored do shed light on the nature of the SCPs if interpreted rigorously. On the one hand, compared to the active movements, passive movements differ in that the CNS does not need to compute the detailed trajectory of the arm. However, neural correlates of proprioceptive sensory feedback are still present. Nevertheless, our results show that passive movements cannot be decoded from SCPs suggesting that there is little influence of proprioceptive feedback in the decoding. The velocity profiles of the movements performed for both conditions are similar suggesting this should not influence the final decoding performance. Shuffle and random conditions show residual correlations which do not yield appropiate trajectory reconstruction and could be again a consequence of the correlation metrics. However, with this small sample size, caution must be applied and further evaluation should be performed using larger datasets.

The decoding accuracies are lower than those reported in a recent work [22], where the authors state that it is possible to accomplish a two-dimensional real time control of a cursor with performance levels comparable to those of invasive BMI systems. In this case, decoding performance is also subject to scaling limitations. For these reasons, we have proposed a simplification of the method by computing a classification of reached targets (discretization of the continuous decoding). This kind of approach has been also explored in several works [16, 31, 32]. In our case, the results have shown high success rates for different target configurations, presenting a clear consistency with the previously obtained decoding performance for continuous movements. These results are quite encouraging and suggest that an online application of this methodology may provide an accurate identification of upper limb movement intention. By reducing the dimensionality of the classification output, this classification approach presents promising advantages in future neurorehabilitation procedures, where EEG slow cortical potentials could be exploited to classify arm movement directions [33] and even detect movement onset [34]. This again corroborates the trajectory-encoding features of the SCPs for the active condition. Regarding rehabilitation assistance, a classification of reached targets may be more suitable as rehabilitation therapy is often based on repetitive movements [35].

In future studies it would be interesting to also assess the role of high-frequency modulations, for instance, by correlating the envelopes of those higher frequency bands to the cursor signal. Recent works by Farina and colleagues have suggested that force generation is mainly due to low-frequency neuromuscular inputs as the neural drive acts as a linear filter that removes any component over 10 Hz [36]. Contrary to this, most of the studies that deal with corticomuscular coherence show that EEG signals are generally coupled with EMG at higher rates (beta and gamma bands), which, apparently, has no functional meaning [37, 38]. One interesting point would be to assess if high-frequency oscillations are modulated at a slower rate and, thus, carry information of functional motor cortical inputs, which could explain those findings in corticomuscular coherence. This behavior of high-frequency cortical components could also explain functional modulations of alpha (8-12 Hz) and beta (16-30 Hz) bands, widely used in classical BCI-based protocols, such as motor imagery. Another interesting point would be the evaluation of which bands provide more information before the movement (planification) and during the movement (execution).

## Conclusion

The main goal of this study was to shed light to the controversy of current decoding procedures. For this reason, we have replicated the same core methodologies of previous studies [17, 21, 22], i. e., multidimensional lineal regression applied to center-out reaching tasks. We have found significant decoding performance when applying these linear decoders to slow cortical potentials (0.1-2 Hz). However, decoding performance is subject to scaling limitations and there is also variability on the decoded trajectory. For this reason, we have proposed a more reliable way of decoding subject’s motor execution from the continuous decoding of trajectories (classification of reached targets) aiming at a future application in a rehabilitation context. Additional control experiments (passive reaching tasks) have been assessed to show that proprioceptive feedback has little influence in the decoding, suggesting that discriminant modulations of low-frequency neural activity are mainly related to the execution of movement.

## Abbreviations

BMI: Brain-machine interface
CNS: Central nervous system
EEG: Electroencephalography
ECoG: Electrocorticography
EMG: Electromyography
FES: Functional electrical stimulation
MEG: Magnetoencephalography
SCI: Spinal cord injury
SCP: Slow cortical potential
